# Pathogenic New World Relapsing Fever *Borrelia* in a *Myotis* Bat, Eastern China, 2015 

**DOI:** 10.3201/eid2612.191450

**Published:** 2020-12

**Authors:** Hui-Ju Han, Jian-Wei Liu, Hong-Ling Wen, Ze-Min Li, Si-Cong Lei, Xiang-Rong Qin, Chuan-Min Zhou, Hao Yu, Xiao Xiao, Xue-Jie Yu

**Affiliations:** Wuhan University, Wuhan, China (H.-J. Han, J.-W. Liu, Z.-M. Li, S.-C. Lei, X.-R. Qin, C.-M. Zhou, H. Yu, X.-J. Yu);; Shandong University, Jinan, China (H.-L. Wen);; Hubei University of Chinese Medicine, Wuhan (X. Xiao)

**Keywords:** Bacteria, bats, *Borrelia*, *Candidatus* Borrelia fainii, China, housekeeping genes, *Myotis* bats, New World relapsing fever, relapsing fever, spirochetes

## Abstract

We identified *Candidatus* Borrelia fainii, a human pathogenic bacterium causing New World relapsing fever in a *Myotis* bat in eastern China. This finding expands knowledge about the geographic distribution of *Borrelia* spp. and the potential for infection with New World relapsing fever in China.

*Borrelia* is a genus comprising 3 groups of spirochetes: the Lyme disease group, the relapsing fever group, and a nonconformist third group. Typically, Lyme disease borreliae are transmitted by hard ticks and have a worldwide distribution, but most relapsing fever *Borrelia* are transmitted by soft ticks, except for louse-borne *B. recurrentis*. Relapsing fever borreliae are further classified into 2 subgroups, New World relapsing fever (NWRF) *Borrelia* and Old World relapsing fever, on the basis of epidemic regions and the genetic lineage of the causative agent. *B. lonestari* and *B. miyamotoi* are transmitted by hard ticks, but are more closely related to relapsing fever borreliae than to Lyme disease borreliae and are distributed both in the New World (North and South America and Oceania) and the Old World (Europe, Asia, and Africa). The nonconformist third group includes an orphan *Borrelia* species named *B. turicata* (*1*). We identified *Candidatus* Borrelia fainii in a *Myotis* bat from eastern China. 

During March–October 2015, with the help of local farmers, we caught a total of 145 bats from various niches in Mengyin County, Shandong Province, China, using mist nets or butterfly nets and took tissue samples (liver, spleen, lung, or kidney). The captured bats included 4 *Rhinolophus ferrumequinum* and 14 *Rh. pusillus* from a karst cave, 26 *Eptesicus serotinus* from 2 farm houses, 34 *Myotis fimbriatus* and 10 *M. ricketti* from a city sewer, and 57 *M. pequinius* from a cave. We extracted DNA from the bat tissue, then screened for *Borrelia* by amplifying the *rrs*, *flaB*, and *glpQ* genes using methods described previously (*2**–**4*); after gel extraction, we cloned the PCR products into pMD19 T-vectors (TaKaRa, https://www.takarabio.com) for sequencing. 

We found 1 *M. ricketti* bat positive for *Borrelia* (GenBank accession nos. MG832412 for *rrs*, MG832413 for *flaB*, and MG921625 for *glpQ*). BLAST searches showed that *rrs* exhibited 99.7% (1,491/1,495 bp) identity with *Candidatus* Borrelia fainii (accession no. LC382043), *flaB* exhibited 97.9% (756/772 bp) identity with *B*. *turicatae* (accession no. CP015629), and *glpQ* exhibited 97.6% (859/880 bp) identity with *B*. *parkeri* (accession no. AY934633). 

We performed multilocus sequence typing (MLST) by amplifying 8 housekeeping genes (*clpA*, *clpX*, *nifS*, *pepX*, *pyrG*, *recG*, *rplB*, and *uvrA*) with degenerate primers from the *Borrelia* MLST database (https://pubmlst.org/borrelia). Sequence query showed that all 8 housekeeping genes were novel alleles, which were assigned the following novel allele numbers: *clpA* (298), *clpX* (261), *nifS* (235), *pepX* (264), *pyrG* (277), *recG* (292), *rplB* (254) and *uvrA* (268); the *Borrelia* species of this study was designated sequence type (ST) 927. 

Sequences of the 8 housekeeping genes were concatenated in the order *clpA*, *clpX*, *nifS*, *pepX*, *pyrG*, *recG*, *rplB*, and *uvrA* and imported into MEGA7 (MEGA, https://www.megasoftware.net) for phylogenetic analysis. We constructed a phylogenetic tree using the maximum-likelihood method with the Kimura 2-parameter model. The ST927 *Borrelia* species was phylogenetically closely related to multiple NWRF *Borrelia* species, including *B. turicatae*, *B. parkerii*, and *Candidatus* Borrelia johnsonii, which are endemic in the United States, as well as *Candidatus* Borrelia fainii, which was recently identified in Zambia (Figure). 

**Figure:**
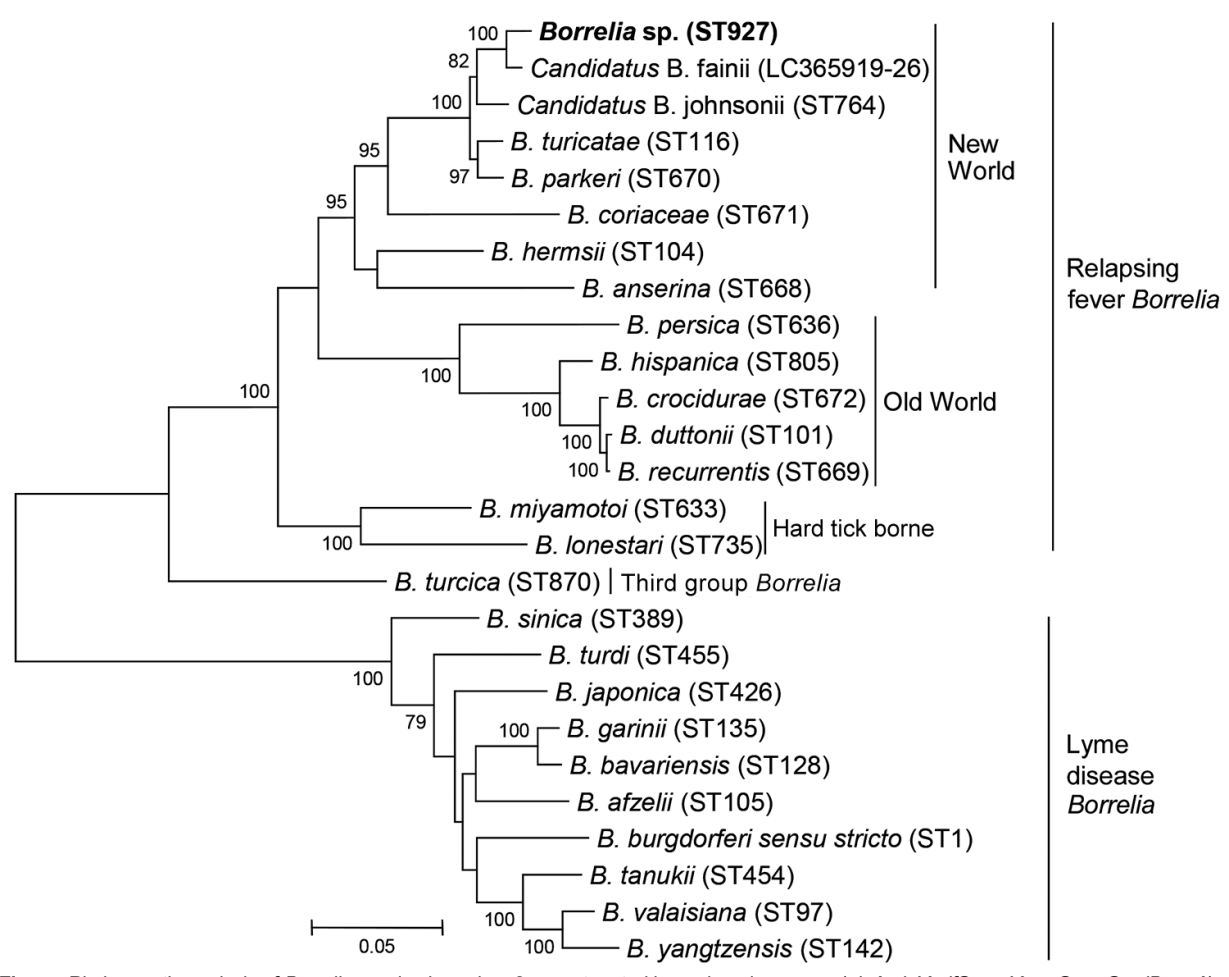
Phylogenetic analysis of *Borrelia* species based on 8 concatenated housekeeping genes (*clpA*-*clpX*-*nifS*-*pepX*-*pyrG*-*recG*-*rplB*-*uvrA*). Bold indicates *Borrelia* species identified in study of pathogenic New World relapsing fever *Borrelia* in a *Myotis* bat, eastern China, 2015. The tree was constructed by using the maximum-likelihood method in MEGA7 (https://www.megasoftware.net). Bootstrap values were calculated with 1,000 replicates. There were a total of 4,776 positions in the final dataset. Reference sequences of *Borrelia* species were downloaded from the *Borrelia* MLST database; the corresponding sequence type (ST) number of each *Borrelia* species is shown in parentheses. For *Candidatus* Borrelia fainii, the GenBank accession number is shown instead of an ST number because the 8 housekeeping gene sequences of *Candidatus* Borrelia fainii were only submitted to GenBank and no ST number was assigned. Scale bar indicates 5% divergence.

We calculated pairwise genetic distances using the Kimura 2-parameter model and identified relapsing fever *Borrelia* spp. using the threshold of 98.3% similarity and genetic distance 0.017 (*5*). Genetic distance analysis of the 8 concatenated housekeeping genes (4,776 bp) revealed a value of 0.015 compared with *Candidatus* Borrelia fainii, strain Qtaro. Thus, the *Borrelia* sp. in our study was identified as *Candidatus* Borrelia fainii. 

There have been several reports of NWRF *Borrelia* spp. in the Old World, although exclusively in Africa. A new human pathogenic *Borrelia* spp. was identified in *Ornithodoros* ticks from Tanzania that grouped together with NWRF borreliae rather than the relapsing fever–inducing spirochetes previously known to be endemic in East Africa (*6*). Another study described the discovery of a NWRF *Borrelia*, *Candidatus* Borrelia kalaharica, in a traveler returning from the Kalahari Desert (*7*). Finally, a 2019 study reported on a NWRF *Borrelia*–like spirochete, *Candidatus* Borrelia fainii, recently isolated from a febrile patient as well as from bats and bat ticks in Zambia (*8*). 

Whether bats are reservoirs for *Borrelia* remains inconclusive. A new Old World relapsing fever *Borrelia* species, CPB1, was found responsible for the death of a *Pipistrelle* bat in the United Kingdom (*9*) and was also detected in bat soft ticks in France (*10*). A recent study found that bats and bat soft ticks collected from a cave in Zambia showed a high infection rate for *Candidatus* Borrelia fainii, and the authors proposed that bats contribute to the environmental cycle of *Candidatus* Borrelia fainii as hosts and bat soft ticks as vectors (*8*). For this study, we found only 1 bat infected with *Candidatus* Borrelia fainii, and it remains unclear whether bats serve as reservoirs of this *Borrelia* species. However, with the discovery of *Candidatus* Borrelia fainii in China, both health officials and physicians should pay attention to its potential emergence. 
